# Predicting the Development of Executive Functions in Preschool Age: Motor, Language, and Socio-Relational Skills in Early Childhood

**DOI:** 10.3390/jintelligence14040054

**Published:** 2026-04-01

**Authors:** Nicoletta Scionti, Claudia Ceruti, Maria Laura Guercio, Gian Marco Marzocchi

**Affiliations:** 1 Department of Psychology, University of Milan-Bicocca, 20126 Milan, Italy; nicoletta.scionti@hotmail.it (N.S.); c.ceruti9@campus.unimib.it (C.C.); marialaura.guercio@gmail.com (M.L.G.); 2 Centro per l’Età Evolutiva, 24121 Bergamo, Italy

**Keywords:** language, motor development, social–relational skills, executive functions, preschoolers

## Abstract

This study investigates the relationship between language, motor, and social–relational development in early childhood and the development of executive functions in a sample of 110 preschoolers (*M* = 57 months, *SD* = 9.8; 47.3% male, 52.7% female). Through the administration of the Preschool Observation of Development and Self-Regulation questionnaire to parents, information about motor, language, and socio-relational skills at 6–36 months and 37–72 months was collected for each participant. Executive functions were investigated by the administration of a neuropsychological battery. The scores obtained on these tests were summarized through confirmatory factor analysis in the two dimensions: working memory–cognitive flexibility and inhibitory control. Multiple regressions were performed to determine whether the development of certain motor, language or social–relational skills had an impact on the development of working memory, flexibility and inhibitory control observed at preschool. The results show that prior language skills, especially grammatical skills, are predictive factors for the development of working memory and cognitive flexibility at preschool. Additionally, some gross-motor skills at 6–36 months are significant predictors for the development of inhibitory control. These skills are therefore clinically important to prevent possible executive impairment in preschool children and to intercept early at-risk children.

## 1. Introduction

Executive Functions (EFs) refer to a set of higher-order cognitive skills that individuals employ to plan and achieve a goal when relying on automatic behavior is not sufficient. These skills are typically conceptualized as three core, interrelated processes: Inhibitory Control (IC), Working Memory (WM), and Cognitive Flexibility (CF) (Miyake et al., 2000). From a developmental perspective, executive functions emerge early in development but follow a prolonged and non-uniform maturational trajectory extending into adolescence (Anderson et al., 2002; Thompson & Steinbeis, 2020). Initial forms of executive control can be observed within the first years of life (Fiske & Holmboe, 2019), while the most rapid developmental changes occur during the preschool period, which represents a sensitive window for executive functioning development (Diamond, 2013). This acceleration is closely linked to the progressive maturation and functional specialization of the prefrontal cortex, whose development continues well beyond childhood (Anderson et al., 2002; Thompson & Steinbeis, 2020). Importantly, distinct executive components show partially dissociable developmental trajectories. IC is among the earliest executive functions to emerge and undergoes substantial improvement during the preschool years (Carlson, 2005; Best & Miller, 2010), whereas WM develops more gradually across childhood and adolescence (Best & Miller, 2010). CF, in contrast, follows a more protracted course, with preschool-aged children typically able to manage simple shifts between tasks or rules, while more complex and unexpected set-shifting abilities continue to mature later in development (Best & Miller, 2010). Consistent with this view, executive development involves both quantitative improvements in efficiency and qualitative changes in the organization of underlying neural systems, with increasing differentiation of executive components over time (Scherf et al., 2006; Best & Miller, 2010). From this perspective, the assessment of executive functioning in preschool age captures emerging and still-developing processes—particularly for CF—rather than fully mature abilities, making early-life predictors especially relevant for understanding individual differences in executive development.

The study of EF is particularly relevant in developmental clinical practice, as early alterations in this system during the preschool period have cascading effects on multiple aspects of life, including academic and occupational success, adaptive functioning, and both mental and physical health (Blair, 2002; Moffitt et al., 2011). From a developmental perspective, these cascading effects can be framed within a developmental cascade model, according to which cognitive functions do not mature in isolation but emerge from dynamic and reciprocal interactions among multiple developmental domains (Karmiloff-Smith, 1998). In this framework, early variations or delays in motor, language, or socio-relational skills are not viewed as deterministic risk factors, but as potential constraints that may alter children’s opportunities for exploration, interaction, and self-regulation across development. Such early differences may therefore shape later executive functioning indirectly, through probabilistic and domain-interactive pathways, rather than through linear or unidirectional mechanisms. Moreover, executive dysfunction appears to play a crucial role in the characterization of several neurodevelopmental disorders (e.g., Attention Deficit Hyperactivity Disorder—ADHD; ASD—autism spectrum disorder; developmental language disorder) and may sometimes exacerbate the clinical expression of pre-existing medical conditions (e.g., phenylketonuria) (Sun & Buys, 2012). In recent years, the literature has highlighted that EFs can be trained and supported through targeted interventions during the developmental window in which they are most malleable, which is the preschool period (Scionti et al., 2020).

For these reasons, identifying early-life predictors of EF development is clinically relevant both in typically developing children and in those with atypical developmental trajectories. Understanding which early competencies and/or experiences most strongly influence later EF development is essential for comprehensive diagnostic assessment and effective intervention planning. Among the potential early risk factors for EF development are delays in motor, language, and social–relational milestones prior to the preschool period. According to a probabilistic and domain-interactive view of development (Karmiloff-Smith, 1998), such delays may impose constraints on other developmental domains, including EF. This hypothesis is indirectly supported by evidence from longitudinal perspective studies on developmental trajectories (Gurevitz et al., 2012; Jaspers et al., 2013) in children with neurodevelopmental disorders, who frequently present with executive dysfunction (e.g., ADHD).

For example, motor-development anomalies observed in the very first months of life—such as an unusually early or delayed achievement of independent sitting and walking—have been frequently reported in children later diagnosed with ADHD (Gurevitz et al., 2012; Jaspers et al., 2013). Similarly, atypical general movements in the postnatal period appear to predict attentional and self-regulation difficulties during the preschool and school years (Hadders-Algra & Groothuis, 1999). There is also direct evidence linking early gross- and fine-motor development to executive functioning in typically developing children. Herbert et al. (2007), for instance, found a positive association between delayed crawling and difficulties in memory retrieval during the first year of life. Associations between motor and executive development in early childhood have also been described by Gottwald et al. (2016). Specifically, 18-month-old children’s ability to modulate the speed of their reaching movements in a reach-and-drop task was positively associated with their performance in memory and inhibition tasks, suggesting that motor control and executive control are interconnected from very early in development. Ridler et al. (2006) similarly identified significant correlations between the timing of independent walking and adult WM performance: individuals who achieved autonomous bipedal gait earlier displayed greater frontal and cerebellar grey matter density and obtained higher scores on memory tasks.

Despite this growing body of evidence indicating that executive control is grounded in action and sensorimotor experience, Pearce and Miller (2025) noted that many influential models of executive function development still do not fully account for the role of movement, thereby underestimating its potential contribution to the emergence of executive abilities during early childhood. Complementing this theoretical perspective, a recent systematic review by van der Veer et al. (2024) reported consistent associations between motor performance and executive functioning in early childhood and showed that the strength of this relationship does not substantially differ between executive tasks requiring motor versus verbal responses. These findings indicate that the motor–EF relationship cannot be explained solely by shared task response demands and emphasize that neither motor performance nor executive functioning should be considered in isolation. Rather, both domains appear to be embedded within broader developmental processes, including general cognitive mechanisms such as processing speed (Roebers & Kauer, 2009).

These empirical findings are also consistent with theoretical accounts suggesting that executive functions are fundamentally grounded in the motor system. According to Koziol and Lutz (2013), the architecture of EF emerges from sensorimotor interactions with the environment, as cognition evolves primarily to regulate action. From this perspective, motor behavior provides the foundation for both procedural and declarative learning processes, which subsequently interact to support the maturation of EFs. On the other hand, some studies suggest that the relationship between motor and executive development may be bidirectional. Emerging executive abilities can themselves act as prerequisites for specific motor learning processes (Bisagno & Morra, 2018), highlighting the reciprocal and dynamic interplay between these domains across early childhood. This convergence of empirical and theoretical evidence is further supported by neurobiological accounts. Motor and executive functions partially rely on overlapping neural circuits involved in action control and executive regulation (Kaiser et al., 2015). Both domains depend on the integrity of thalamic pathways, which integrate information across pallido–thalamo–cortical and cerebello–thalamo–cortical loops. Alterations in these circuits have been documented in children with ADHD, who typically show reduced performance in executive tasks (Valera et al., 2007). Together, these findings indicate that the motor and executive systems are intimately connected at behavioral, developmental, and neural levels.

The association between delays in language development and EF deficits is well documented in the literature, although the direction of this relationship across development remains uncertain. Indeed, despite substantial evidence of an association between language and executive functioning, only a limited number of studies have explicitly examined the directionality of their developmental pathways (Shokrkon & Nicoladis, 2022). Significant impairments in attention and EF have been widely described in children with developmental language disorders (Petersen et al., 2013; Vallotton & Ayoub, 2011) compared to peers with typically developing language skills (Henry et al., 2012; Im-Bolter et al., 2006). According to some authors, this association may reflect an underlying neural processing deficit that not only hinders language acquisition but also affects performance on executive tasks, as suggested by studies showing distinct patterns of event-related potentials in children with atypical language development compared to controls (Sanders et al., 2006).

Multiple lines of research indicate that executive functions and language abilities are overlapping developmental processes. First, studies have shown that early language development relies on executive processes (Sanders et al., 2006; Verhagen & Leseman, 2016). Most of this work has focused on the role of WM in language acquisition, demonstrating, for example, that children’s ability to update information in WM contributes to vocabulary growth (Weiland et al., 2014) and to lexical learning and organization processes (Morra & Camba, 2009). Second, several authors have argued that language supports the development of EF through the emergence of inner speech (Montgomery et al., 2010), which children initially use in explicit, private form to regulate and plan goal-directed actions (Vygotsky, 1962). Third, initial evidence suggests that interventions targeting EF can improve preschoolers’ language abilities (Fuhs & Day, 2011; Petersen et al., 2013). Recent findings further indicate that language interventions that modulate executive-function demands may enhance language growth even in children with weaker EF skills (Rojas-Barahona et al., 2015). Beyond reciprocal and overlapping developmental processes, some authors have suggested that the association between language and executive functioning may also reflect shared underlying risk factors. For example, Bishop et al. (2014) proposed that common genetic or neurobiological vulnerabilities—such as atypical development of frontal brain regions—may concurrently affect neural systems supporting both language and executive control.

More recently, longitudinal and cross-lagged studies have demonstrated a bidirectional and reciprocal relationship between language and executive development, depending on the developmental period and on the specific linguistic and executive components examined (Slot & von Suchodoletz, 2018; Viterbori et al., 2012). Inhibitory abilities at 24 months appear to predict phonological accuracy and communicative intelligibility at 36 months, whereas morphological and syntactic skills—such as the appropriate use of inflected forms of verbs, nouns, and adjectives—predict later CF and more complex inhibitory processes (Viterbori et al., 2012). Bohlmann et al. (2015) further found a reciprocal association between language and EF from only around 56–62 months of age, with EF exerting a stronger influence on lexical skills at earlier stages. Consistent with this view, recent integrative reviews have emphasized that evidence supports multiple, non-mutually exclusive developmental pathways between language and executive functions, including EF-to-language, language-to-EF, bidirectional, and shared-factor models, with their relative contribution varying across developmental stages (Shokrkon & Nicoladis, 2022). Converging behavioral and neuroimaging evidence further supports a bidirectional and developmentally contingent interaction between language and executive functioning across childhood, highlighting partially overlapping neural networks underlying these processes (Pu et al., 2025).

Regarding socio-relational development, several studies have reported an association between executive and social–relational competencies (Welsh et al., 2010; Weissberg et al., 1989). Children with stronger executive abilities are generally more capable of adjusting and integrating behaviors, actions, and emotions that are necessary for managing social tasks (Weissberg et al., 1989), and they tend to display higher levels of social and emotional competence. Cole et al. (1993) showed that difficulties in EF, which were positively correlated with preschoolers’ inability to regulate disruptive behavior, had both direct and indirect consequences on socio-relational skills. Longitudinal studies further indicate that children with stronger EF abilities are better positioned to establish and maintain positive peer relationships compared to peers with weaker executive skills (Welsh et al., 2010). Moreover, foundational milestones of socio-relational development, such as the emergence of theory of mind, are known to be closely linked to the development of EF, particularly IC (Carlson et al., 2015). Recent meta-analytic evidence strengthens this picture; a large-scale meta-analysis synthesizing 1459 effect sizes from 158 studies found that early childhood EF (36–60 months) is consistently associated with several socio-relational outcomes, both concurrently and longitudinally. Specifically, EF was positively related to emotion understanding, emotion regulation, prosocial behavior, peer acceptance, and social competence, and negatively related to internalizing problems, externalizing behavior, inattention, and hyperactivity. These findings indicate that EF contributes broadly to children’s social adjustment and behavioral functioning (Stucke & Doebel, 2024).

Less is known, however, about how early socio-relational milestones contribute to later EF development. Through their first interactions with primary caregivers, children learn how to engage in relationships, how to have their needs and desires met, and how to identify and regulate emotions. Early family and caregiving experiences appear to support EF development in ways that are still being clarified. For example, joint attention processes, parental emotional responsiveness, scaffolding, and parental behavioral modeling can facilitate the development of self-regulation, autonomy, and flexible behavior (Barrasso-Catanzaro & Eslinger, 2016). Over time, turn-taking, sharing, the introduction of problem-solving strategies, and experiences with reward contingencies introduce additional foundational components of EF (Bernier et al., 2010). In supportive caregiving environments, these experiences interact with the child’s neurobiological potential to foster the emergence of highly adaptive capacities. Although the mechanisms underlying this association remain unclear, early experience—particularly within the family and caregiving context—has been identified as a fundamental foundation for the later maturation of EF. Conversely, early adversity linked to poverty or psychosocial deprivation can disrupt these foundational components and negatively affect EF development (Barrasso-Catanzaro & Eslinger, 2016). Recent work has examined how early caregiving interacts with socioeconomic and environmental adversity; although positive parenting relationships did not fully buffer the impact of these adversities on children’s EFs and socioemotional skills, parenting quality emerged as a stronger correlate of children’s everyday EF and socioemotional competence than either socioeconomic status or household chaos alone (McDorman et al., 2025). These findings underscore the importance of early caregiving environments for EF maturation, even in the context of elevated risk.

### The Present Study

In this study, we investigated the relationship between early language, motor, and socio-relational development across different developmental windows and the emergence of EF in preschool age. Drawing on the developmental cascade model (Karmiloff-Smith, 1998), we hypothesized that delays in the achievement of key motor, language or socio-relational milestones during early childhood (6–36 months) may influence later EF development in preschoolers. Within this developmental cascade framework, early delays in motor, language, and socio-relational domains are expected to influence later executive functioning through cascading effects that depend on their timing and persistence across developmental windows; the present study was designed to examine this process in preschool age. By adopting this approach, the present study contributes to current models of executive function development by specifying how early domain-specific and time-dependent developmental variations may differentially shape distinct components of executive functioning in preschool age. Indeed, we examined which early developmental milestones were most predictive of EF development and whether these early influences persisted into preschool age, independently or in interaction with later emerging risks. Although theoretical models emphasize potential reciprocal influences between EFs and early developmental domains, the present study adopts a predictive framework. Specifically, early developmental milestones (6–36 months) were treated as antecedent indicators of later EF performance in preschool age. Given the retrospective assessment of early milestones and the single time-point measurement of EF, the design does not allow for testing bidirectional effects.

In addition to describing children’s developmental trajectories across motor, language, and socio-relational domains, the study had two main aims. First, we sought to identify the latent structure of EF in our preschool sample, in order to derive robust EF dimensions for subsequent analyses. Second, we aimed to determine whether delays in motor, language, or socio-relational milestones in early childhood predicted preschool EF, and whether the persistence or resolution of such delays in the preschool years further shaped these outcomes. This approach allowed us to investigate how different developmental pathways, emerging at distinct moments in early childhood, may contribute to the emergence of EF in preschool age.

These objectives address several gaps in previous research, which has often examined early developmental predictors of EF either within single domains, in clinical samples, or without explicitly considering the timing and persistence of early delays. Moreover, given the transdiagnostic relevance of executive functions across multiple neurodevelopmental disorders and their documented malleability during the preschool period, identifying early developmental pathways associated with EF may have important implications for early monitoring and preventive intervention.

## 2. Materials and Methods

### 2.1. Participant

A total of 110 children participated in the study (52 boys and 58 girls), aged between 3 and 6 years (*M* = 57 months; *SD* = 9.8). All children were attending public or private preschools in the Lombardy region, in the province of Milan. The socioeconomic status of participating families fell within the middle-to-high range (mothers: 56% technical/clerical sector, 18% managerial, 10% crafts, 13% unemployed; fathers: 42% technical/clerical sector, 30% managerial, 19% crafts, 9% unemployed). Regarding background characteristics, information on race/ethnicity was not systematically collected. Concerning language, 88.2% of the sample were native Italian speakers. The remaining 11.8% had been residing in Italy for 1–6 years and demonstrated adequate comprehension of the task instructions during assessment.

Participants were selected from an initial sample of 416 children. Children scoring below the 15th percentile on the Raven’s Progressive Matrices and/or those with more than 10% missing responses on the developmental questionnaire were excluded. From the remaining 298 children, those who scored below the 15th age- and/or gender-corrected percentile on one (*n* = 33) or both (*n* = 24) of the two global scales of the Preschool Observation of Development and Self-Regulation Questionnaire (PODS-Q) (“Development in the first three years of life” and “Current developmental level”) were included in the risk group (*n at risk* = 57; females = 26). To obtain a statistically comparable control group with similar sample size, approximately 22% of the remaining children (*n_non-risk_* = 53; females = 32) who scored above the 16th age- and/or gender-corrected percentile on both global scales were randomly selected. The two groups did not differ significantly in gender distribution (χ^2^(1) = 2.40, *p* = .121) or age (χ^2^(2) = 1.81, *p* = .405). Table 1 summarizes the stratification of the final sample included in the study (*N* = 110). Specifically, crossing the retrospective early developmental risk classification (6–36 months) with the preschool classification (3–6 years) yielded four subgroups: 24 children at risk at both time points, 29 at risk only at 6–36 months, 4 at risk only at 3–6 years, and 53 not at risk at either time point. Importantly, the term “risk” refers to scoring below the 15th age- and/or gender-corrected percentile on the PODS-Q global scales and does not indicate a clinical diagnosis; at the time of data collection, none of the participating children had received a formal neurodevelopmental diagnosis.

The four subgroups did not differ significantly in sex distribution (χ^2^(3) = 3.11, *p* = .374), age (*F*(3, 16.5) = 2.93, *p* = .064), or Raven percentile scores (*F*(3, 13.9) = 1.53, *p* = .250).

### 2.2. Procedure

Participants were recruited through preschools, either contacted directly or through the municipal “Education Directorate” of the city of Milan. Data collection took place in 2019/2020 after obtaining authorization from school principals and after parents provided written informed consent. Following consent, the study included the individual assessment of the children and the collection of developmental information from their parents.

The assessment battery consisted of six tasks administered individually to the children in a fixed order, as listed in Section 2.3, during school hours in a quiet room specifically prepared for the sessions. In each preschool, the room was selected among the available spaces to ensure minimal distractions and adequate quiet conditions for task administration. Although the specific physical characteristics of the rooms varied across schools, efforts were made to maintain comparable testing conditions in terms of noise level and absence of external interruptions. The battery was administered in a single session lasting on average 30 min by four master’s-level graduates adequately trained to administer the tasks and blinded to the child’s risk group assignment. All protocols were labeled with a unique alphanumeric code to ensure participant anonymity.

Consistent with what frequently occurs in clinical settings, retrospective developmental information was collected through a parent-report questionnaire in which caregivers indicated whether their child did or did not display specific skills within given age ranges (e.g., whether at 6 months the child “called for parents using specific sounds,” “sat without support,” or “showed affection through hugs or kisses”). The questionnaire was distributed in paper format to parents through the preschools during the data collection period (2019–2020). Caregivers completed the questionnaire at home and returned it to the school once filled in, and no specific deadline was imposed. Similarly, information regarding children’s language, motor, and socio-relational abilities during the preschool period (37–72 months) was gathered to obtain an overview of their current developmental profile and to trace an individual developmental trajectory across the domains of interest.

The study was conducted in accordance with the ethical standards of the Declaration of Helsinki and was approved by the Ethics Committee of the University of Milan-Bicocca (protocol code 534/date of approval 9 July 2020).

### 2.3. Measures

The six tasks included in the assessment battery are described below.

Shape and Color Game: The task, taken from the Preschool Executive Function Assessment Battery 2–6 (FE-PS 2–6 Battery) (Usai et al., 2017), includes three conditions that assess children’s cognitive flexibility, namely their ability to alternate between tasks and different response sets. The examiner asks the child to categorize cards depicting a red rabbit and a blue boat first by color (first condition), then by shape (second condition), and finally to shift between the two rules based on the presence or absence of a black border on the card (third condition). The maximum score is 24 points (6 for the first condition, 6 for the second, and 12 for the third).

Day and Night Game: This task is a variant of the Stroop-like task from the FE-PS 2–6 Battery (Usai et al., 2017) and it assesses inhibitory control. It consists of two phases: a baseline phase, in which the child must say what is depicted on the card (a sun or a moon), and a Stroop phase, in which the child must say the opposite of what is shown. The maximum score is 36 points and reflects accuracy across the two phases.

Truck Loading: This task is a variant of the original paradigm developed by Fagot and Gauvain (1997). The examiner presents the child with a model of a street lined with houses of different colors, to which letters of matching colors must be delivered. Since the street can be traveled in only one direction, the child must preload the letters in the correct order into a small truck used for delivery. The task assesses planning abilities and WM. It consists of four levels, each with two trials. At each level, the number of houses increases by one, from a minimum of two to a maximum of five. The maximum score is 8 points and reflects accuracy across trials.

Statue: Taken from the NEPSY-II battery (Korkman et al., 2007), this task assesses motor inhibition and motor persistence. The child is asked to maintain a specific posture with eyes closed for 75 s while inhibiting the impulse to respond to auditory distractions produced by the examiner. The score (0–45) reflects the number of errors made during the 75 s (movements, vocalizations, or opening the eyes). Raw scores were used to ensure consistency and comparability with the other executive function raw measures included in the analyses.

Digit Span: Taken from the Wechsler Intelligence Scale for Children–IV (Wechsler, 2012), this task assesses the forward and backward span of WM. In the forward condition, the task measures short-term storage processes, whereas in the backward condition it assesses the active component of WM (i.e., the child’s ability to mentally manipulate information). The task required only auditory repetition of number sequences and did not involve numerical calculation or symbolic processing, thereby minimizing potential confounding effects of number knowledge for the youngest participants. The maximum score for each condition is 16; raw scores were used, and consistent with methodological recommendations (Conway et al., 2005), scoring was based on the total number of correctly recalled sequences (0/1 scoring) rather than on absolute span scores, in order to maximize sensitivity to individual differences, including among younger children.

The Gift: This task is based on the delay aversion paradigm and is a variant of the task proposed by Sonuga-Barke et al. (1992) and it assesses delay of gratification and inhibitory control in a motivational context. In this version, the examiner presents the child with a box containing a small gift and asks whether they prefer to receive the gift immediately or wait two minutes to obtain a second one. The score corresponds to the maximum waiting time tolerated by the child (0–120 s).

Preschool Observation of Development and Self-Regulation Questionnaire (PODS-Q; Conti et al., 2020): It is an observational questionnaire, validated in Italy and completed by parents of preschool children, designed to outline a child’s developmental profile from the early years of life to the present and to identify possible significant difficulties in self-regulation. The questionnaire consists of 90 items and is composed of two parts.

The First Part includes three empirically derived subscales (Conti et al., 2020): Gross and Fine Motor Skills (6–36 months), Language Skills (6–36 months), and Socio-relational Skills and Autonomies (6–36 months). These subscales contain a total of 41 items referring to the child’s development during the first three years of life in the motor domain (13 items), language domain (14 items), and socio-relational/adaptive domain (14 items). Specifically, the behaviors described in this First Part include fundamental milestones of typical development. For the motor domain, examples include sitting without support at six months, walking independently between one and two years of age, scribbling with crayons or pencils, or standing on one foot for a few seconds without support by the third year of life. Similarly, the language domain includes behaviors commonly present in young children’s repertoires, such as calling “mum” or “dad” in the first year, greeting with “hello” or similar expressions in the second year, the appropriate use of definite and indefinite articles when describing objects (e.g., “He used the articles “a” and “the”, for example: “look, a dog”, “look, the kitten””), or the ability to name body parts during the third year. Socio-relational skills include typical behaviors such as showing affection during the first year of life by giving hugs or kisses to a caregiver, showing sympathy toward peers by trying to help them between one and two years, and engaging in group pretend play by the third year. Alongside socio-relational skills, this domain also encompasses autonomies typically acquired during the first three years, such as eating independently, removing socks without help, or unscrewing and screwing the caps of jars and bottles. In this First Part, parents are asked—via a 3-point Likert scale (0 = “no”; 1 = “partly”; 2 = “yes”; “N.R.” = “I don’t remember”)—to recall their child’s abilities at different developmental stages and indicate whether the child did or did not display the behaviors listed in the corresponding periods. As the First Part relies on retrospective parent report of early developmental milestones, potential recall bias should be considered. However, the items refer to salient and developmentally meaningful milestones (e.g., walking, first words, early social behaviors), which are typically more robust to memory inaccuracies than subtle behavioral characteristics.

The Second Part of the PODS-Q includes four subscales (15 items) referring to attentional, behavioral, and emotional self-regulation difficulties—which were not analyzed for the purposes of the present study—and four subscales assessing the child’s current developmental level (34 items): Gross and Fine Motor Skills (3–5 years), Language Skills and Early Academic Prerequisites (3–5 years), Socio-relational Skills (3–5 years), and Autonomies (3–5 years). These subscales allow for an observation of the child’s developmental profile in the motor domain (8 items), language and early academic-prerequisite domain (14 items), socio-relational domain (6 items), and autonomy domain (6 items). The behaviors included in this section cover fundamental aspects of development in this age range, such as the maturation of fine motor skills and eye–hand coordination (e.g., drawing two intersecting lines; coloring within boundaries); the acquisition of early academic prerequisites, such as counting three or more objects; the development of linguistic abilities, such as correctly using the words “today,” “yesterday,” and “tomorrow”; socio-relational skills, such as following simple rules during play or being able to apologize; and aspects of autonomy, such as dressing and undressing independently, washing oneself, or taking care of one’s belongings—although these last items were not included in the aims of the present study. In this Second Part, parents are asked to indicate whether the behaviors listed are present in their child’s current repertoire using a 3-point Likert scale (0 = “no”; 1 = “partly”; 2 = “yes”).

For the calculation of PODS-Q subscale totals, the item scores within each subscale are summed. The First Part of the questionnaire also provides a global score referring to Development in the first three years of life (0–82), whereas the Second Part yields a global score representing Current developmental level (0–68). Higher scores indicate a higher developmental level within each subscale, while scores falling below the 15th percentile are considered within the risk range. In the case of missing responses (“missing” or “N.R.”), it is possible to compute and interpret the subscale total using the formula: *sum of item scores///number of valid responses × total number of items in the subscale*, while avoiding interpretation whenever more than 10% of responses are missing. Raw scores can be converted into gender-corrected percentiles for the First Part, and into age- and gender-corrected percentiles for the Second Part.

### 2.4. Statistical Analyses

To examine the influence of early motor, language, and socio-relational development on executive functioning, we first worked on the dependent variables (i.e., performance on the EF tasks) with the aim of reducing them quantitatively and rendering them continuous. For this purpose, we used confirmatory factor analysis (CFA), which allows for the estimation and comparison of different models of executive functioning within the sample and has the advantage of isolating the shared variance among the observed variables—namely, the scores obtained on the administered EF tasks—thus partially addressing the well-known issue of task impurity in EF measures (Miyake et al., 2000). A prerequisite for applying CFA is the normality of the observed variables, which was assessed by examining skewness and kurtosis indices for each administered task. The confirmatory factor analysis was conducted on the full screened sample (*N* = 298), consistent with our previous validation study (Scionti & Marzocchi, 2021). The resulting factor structure was then applied to the final subsample (*N* = 110) for the computation of the regression-based factor scores used in the predictive analyses.

In line with the literature on the structure of EF in preschool age (Usai et al., 2014; Wiebe et al., 2011; Monette et al., 2015), one-factor (executive control), two-factor (WM–CF and IC), and three-factor models (WM, CF, and IC) were estimated and compared. To determine which of the estimated models showed the best fit, the χ^2^ statistic and the Bentler indices—Root Mean Square Residual (RMSR), Tucker–Lewis Index (TLI), and Comparative Fit Index (CFI)—were examined. Following the criteria reported in the literature, the model with the most adequate fit indices was selected (a χ^2^ statistic close to zero and *p* > .05; RMSEA ≤ 0.06; CFI and TLI ≥ 0.90; Hu & Bentler, 1999).

Factor scores (Z-scores with a mean of 0 and standard deviation of 1) for the dimensions of the best-fitting model were then estimated using a regression-based approach. These factor scores were used as dependent variables in the subsequent analyses, conducted to determine the degree to which the developmental milestones described above predicted the neuropsychological variables. These analyses were carried out on a sample of children (*N* = 110) divided into four groups for each type of developmental risk (motor, language, socio-relational):

- Group 1 (*n* = 29): Children who showed a risk score in the 6–36 months period, which later normalized in the preschool period (3–6 years).
- Group 2 (*n* = 24): Children who showed a risk score in the 6–36 months period, and continued to show a risk score in the preschool period.
- Group 3 (*n* = 4): Children who did not show a risk score in the 6–36 months period but showed a risk score in the preschool period.
- Group 4 (*n* = 53): Children who did not show risk scores in any of the developmental windows considered.

First, a Multivariate Analysis of Variance (MANOVA) was conducted, with the EF dimensions extracted through the CFA (IC and WM–CF) as dependent variables. Independent variables were three dichotomous indicators of early developmental risk at 6–36 months (motor, language, and socio-relational risk), defined as scoring below the 15th age- and/or gender-corrected percentile on the corresponding PODS-Q domain. Main effects and interaction effects were examined to determine (a) which type of risk (motor vs. language vs. socio-relational) within this early developmental window (6–36 months) exerted the strongest influence on EF development in preschool age, and (b) whether the interaction between multiple types of risk had an amplified impact on EF outcomes.

Subsequently, regression analyses were carried out to determine the impact of each type of risk at 6–36 months (early risk) on each EF dimension, and to examine whether this impact varied depending on the persistence or absence of the same developmental risk during the preschool period (late risk). For each developmental domain, mean differences in IC and WM–CF were compared across four groups: children who showed early risk at 6–36 months but no longer showed risk in the preschool period; children in whom early risk at 6–36 months persisted into preschool age; children who showed no early risk at 6–36 months nor late risk at 3–6 years; and children who did not show early risk at 6–36 months but exhibited late risk in the same domain at 3–6 years.

Data were analyzed using IBM SPSS 29.0 (SPSS, Chicago, IL, USA) and Jamovi 2.5.5 (The Jamovi Project, 2021). The alpha level was set at 0.05 for all statistical tests.

## 3. Results

### 3.1. Descriptive Statistics and Analysis of Performance on Executive Function Tasks

Table 2 reports the skewness and kurtosis indices for the executive tasks administered. The analyses showed that all EF measures were approximately normally distributed, with the exception of the Statue task, whose distribution displayed a leptokurtic pattern. However, following Kline (2011), this deviation was considered negligible, as it was not likely to produce misspecification errors (i.e., distortion of parameter estimates) for model estimation and factor reduction. For this reason, a CFA with maximum-likelihood estimation was conducted.

**Figure 1:**
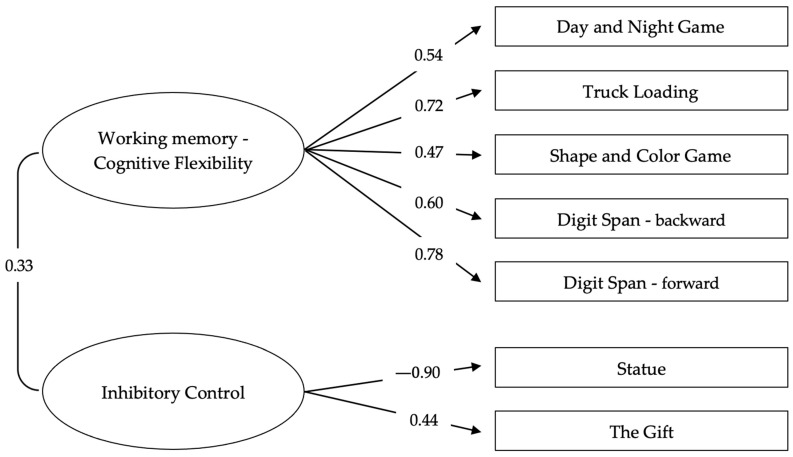
The figure displays the standardized factor loadings on the unidirectional paths and the correlation between the two latent dimensions; these indices derive from the confirmatory factor analysis conducted on performance across the seven executive tasks (see Section 2).

The results indicated that the model with the most adequate fit was the two-factor model, in which WM–CF and IC emerged as distinct yet significantly correlated dimensions [χ^2^ (13, *N* = 110) = 8.85, *p* = .784; CFI = 1.00; TLI = 1.05; RMSEA < 0.001, 95% RMSEA CI: 0.000–0.063]. This factor structure is illustrated in Figure 1. The results are consistent with a previous study (Scionti & Marzocchi, 2021), which included part of the present sample.

### 3.2. Predictive Models of Motor, Language, and Socio-Relational Skills on EF Development

To examine which early motor, language, and socio-relational skills most strongly influenced the EF development observed in preschool age, a MANOVA was conducted. The analysis revealed a significant main effect of early motor development on IC [*F*(1, 102) = 7.29, *p* = .008] and a significant main effect of early language development on WM–CF [*F*(1, 102) = 10.11, *p* = .002]. Specifically, children who showed a critical score in the motor domain during the 6–36 months period obtained lower mean scores on IC tasks compared to children who displayed age-appropriate motor skills in early infancy. Likewise, children who showed a probable delay in the language domain at 6–36 months obtained lower mean scores on WM–CF tasks.

Analysis of the interaction among early motor, language, and socio-relational competencies (6–36 months) revealed no significant effects. This suggests that the influence of a probable delay in one of the domains examined (e.g., language) on EF development does not vary according to the presence of additional risks in other domains (e.g., motor, socio-relational) during the same developmental window, but is independent from them. For this reason, to investigate the impact of early developmental delays—whether resolved or persistent over time—on the EF measures collected in preschool age, the three developmental domains were examined separately using multiple regression analyses.

As shown in Table 3, the multiple regression analyses yielded several significant results. Specifically, when examining the impact of early motor development on WM–CF and IC, a significant relationship emerged between the presence of early motor risk (6–36 months) and inhibitory abilities [*F*(1, 109) = 6.601, *p* = .012; η^2^ = 0.059; R^2^ = 0.076; β = −0.276], whereas no significant association was found between early motor risk and WM–CF [*F*(1, 109) = 1.93, *p* = .168, η^2^ = 0.018; R^2^ = 0.030; β = −0.120]. The interaction between early motor risk and late motor risk was not significant for either WM–CF [*F*(1, 109) = 0.40, *p* = .531] or IC [*F*(1, 110) = 0.16, *p* = .686]. Thus, the effect of early motor risk on inhibitory abilities does not vary depending on whether the same risk persists or resolves during the preschool years; rather, it remains significant independently of late motor risk. Although these interaction effects were not statistically significant, inspection of the means (expressed as Z-scores, *M* = 0, *SD* = 1) across the four groups—classified according to the presence or absence of motor delay in the two developmental windows—suggested descriptive differences across developmental profiles. Children with persistent motor risk (both early and late) obtained below-average scores on WM–CF tasks (*M* = −0.42), compared to those whose early motor risk resolved by preschool age, who showed scores closer to the mean (*M* = −0.07). This pattern was not reflected in IC, for which children showing early motor risk performed below average regardless of whether such risk persisted (*M* = −0.22) or resolved (*M* = −0.11), compared to peers with no early motor difficulties. Among children without early motor difficulties (6–36 months), those who showed poor motor skills in preschool exhibited lower performance on WM–CF tasks (*M* = −0.17). This pattern was not observed for IC, for which preschool motor skills did not appear to be associated with performance (*M* = 0.04). Children who presented no early or late motor risk showed mean-level performance on both WM (*M* = 0.03) and IC tasks (*M* = 0.09).

With regard to the impact of early language development on EF in preschool age, the regression analyses showed a significant relationship between the presence of early language risk and WM–CF [*F*(1, 109) = 6.90, *p* = .010, η^2^ = 0.061; R^2^ = 0.095; β = −0.31]. In contrast, the impact of early language skills on IC was not statistically significant [*F*(1, 109) = 3.14, *p* = .079, η^2^ = 0.029; R^2^ = 0.065; β = −0.255]. Furthermore, the interaction between early and late language risk was not significant for either WM–CF [*F*(1, 109) = 0.20, *p* = .659] or IC [*F*(1, 109) = 0.26, *p* = .612]. In other words, whether a language delay persists or resolves during the preschool period does not significantly influence EF outcomes among children identified as at risk in early infancy. Regardless of preschool-age language skills, children with early language delay show greater difficulties in WM–CF. Although the interaction effects were not statistically significant, descriptive differences were observed across groups. Children who exhibited probable language delay at 6–36 months and continued to display language difficulties during the preschool period showed below-average performance on WM–CF tasks (*M* = −0.49), more so than on IC (*M* = −0.13). Even among those whose early language difficulties resolved by preschool age, performance on WM–CF (*M* = −0.17) remained lower than that observed for IC (*M* = −0.10). Children who did not present early language risk (6–36 months) but showed language difficulties in preschool age scored near the mean on IC (M = −0.02) and slightly below the mean on WM–CF (*M* = −0.10). Finally, children who presented no language risk in either developmental window performed at average levels on both WM tasks (*M* = 0.10) and IC (*M* = 0.11).

When examining the relationship between socio-relational development and executive components, the regression analyses did not yield statistically significant results. The presence of early socio-relational difficulties did not predict children’s performance on WM–CF tasks [*F*(1, 109) = 2.06, *p* = .154, η^2^ = 0.019; R^2^ = 0.026; β = −0.122] or on IC tasks [*F*(1, 109) = 1.30, *p* = .257, η^2^ = 0.012; R^2^ = 0.025; β = −0.155]. Similarly, socio-relational difficulties in the later developmental window did not show effects on WM–CF [*F*(1, 109) = 0.14, *p* = .708, η^2^ = 0.001; R^2^ = 0.026; β = −0.071] or on IC [*F*(1, 109) = 0.02, *p* = .895, η^2^ = 0.000; R^2^ = 0.025; β = −0.011]. Moreover, the interaction between early and late socio-relational risk was not statistically significant for either WM [*F*(1, 109) = 0.63, *p* = .430] or IC [*F*(1, 109) = 0.01, *p* = .935]. Although these effects were not statistically significant, descriptive group differences were observed. Children identified by the questionnaire as having early socio-relational risk and who continued to show socio-relational difficulties in the preschool period displayed lower scores on WM–CF tasks (*M* = −0.26) compared to those whose early difficulties resolved by the later window (*M* = −0.10). This pattern was not observed for IC, where children, regardless of the presence or absence of risk across developmental windows, generally showed average performance.

## 4. Discussion

Understanding which early developmental experiences are associated with later executive functioning is crucial for both research and clinical practice. The present findings indicate that specific motor and language milestones during the first years of life are meaningfully linked to executive outcomes in preschool age within a community sample of typically developing children.

The data collected in this study indicate that early motor difficulties were significantly associated with IC in preschool age, but not on WM. In contrast, motor difficulties emerging later in development appear to be more strongly associated with WM and CF, but not with inhibition. A closer qualitative inspection of the data showed that most children in our sample who scored low on WM tasks specifically displayed weaker fine-motor skills. Parents of these children more frequently reported difficulties in copying two intersecting lines, drawing complete human figures (including head, facial features, arms, body, legs, and feet), and cutting paper from one side to the other with scissors—tasks that require fine-motor coordination and manual dexterity. Conversely, children who scored lower on tasks measuring IC showed, according to parental reports, early deficits in gross-motor abilities: at 6–12 months, they were unable to walk while holding onto furniture or a crib; between 1 and 2 years, they were unable to descend stairs independently or run without falling. These findings are consistent with the literature on atypical development (Johnson et al., 2015; Gurevitz et al., 2012; Athanasiadou et al., 2020), which reports significant correlations between delays in gross and fine-motor development and difficulties in memory and inhibition. Several hypotheses may explain this relationship. Exploration and stimulation are fundamental components of optimal cognitive development. One interpretation of the association between early gross-motor development and IC concerns the reduced opportunities for environmental exploration typically experienced by children with difficulties or delays in movement and locomotion, compared to their motorically competent peers (Libertus & Hauf, 2017). Gottwald et al. (2016) further argue that EF development is embodied and rooted in motor control abilities, particularly in the child’s capacity to plan goal-directed movements (e.g., reaching for an object). The development of WM and CF may be partly supported by the fine-motor skills that emerge during the preschool years, when manual dexterity is systematically trained through drawing and similar activities (Cameron et al., 2012; Bender & Beller, 2012). It is well known that through the body—particularly the hands and fingers—children learn and consolidate representational systems such as the mental number line (Wood & Fischer, 2008) and skills such as counting (Alibali & DiRusso, 1999), which rely on executive processes. Moreover, several studies have shown that training the body yields benefits on EF development (Best, 2010). Although the mechanisms linking motor and executive development are not yet fully understood, one hypothesis proposed to explain this association is that of co-activation: the neural areas involved in both processes partially overlap (e.g., dorsolateral prefrontal cortex, cerebellum; Diamond, 2000).

Language development represents another fundamental aspect of human development to be considered as a developmental domain strongly associated with EF. The results of this study revealed a significant association between early language delay and performance in WM and CF. A closer qualitative inspection showed that children with greater difficulties in these EF components were those who, according to parental reports, more frequently than their peers failed at 1–2 years of age to correctly use the words “me”, “mine”, and “I” and at 2–3 years struggled to correctly use the definite and indefinite articles “a/an” and “the” or to describe objects with sufficient specificity (e.g., “the doll has hair, a dress…”). Parents also reported concurrent difficulties in language and early academic skills during the preschool period: children who scored below average on WM and CF tasks were more likely than peers to misuse temporal terms such as “today”, “yesterday”, and “tomorrow”, and were unable to correctly write their own name in uppercase print. Indicators of delay in morpho-syntactic development such as these therefore appear to be associated with WM and CF performance in preschool age. They may represent early warning signs to consider when identifying potential executive difficulties. It is important to note, however, that this result does not imply that grammatical skills in this developmental window unidirectionally influence WM. It is equally plausible that the very early language weaknesses associated with later EF performance are themselves dependent on the WM capacities that develop during the early years. This interpretation is consistent with numerous studies showing the active role of WM in language acquisition and in the learning of verbal sequences, lexical items, and syntactic rules—processes that rely on WM both for short-term maintenance of information and for long-term consolidation through rehearsal (Archibald & Gathercole, 2006; Viterbori et al., 2012).

When compared with motor development, the achievement of certain early linguistic milestones appears to be the strongest association with WM and CF development in preschool age. As such, observing and monitoring early language development may be particularly relevant for the early identification of children who may later show executive vulnerabilities. It is important to note that the associations observed could partially be explained by the verbal component involved in some of the executive tasks administered. However, because the linguistic demands of several tasks were minimal (e.g., the Truck Loading task) and these tasks were highly representative of the WM-CF dimension, it is plausible that the associations found do not simply reflect task-related verbal demands. Instead, they likely represent the shared variance underlying the different measures of WM and CF.

Although the literature (Ciairano et al., 2007) has partly demonstrated associations between EF development and various socio-relational skills (e.g., theory of mind, cooperation, prosocial behavior), the socio-relational risk factor in the two developmental windows examined was not significantly associated with EF performance in preschool age. This may partly reflect the specific construct assessed by the PODS-Q subscale, which captures socio-relational competence from an adaptive-behavior perspective. In addition, evidence suggests that tasks involving “hot” executive processes are more strongly associated with emotional and social competence (Eisenberg et al., 2000; Rothbart & Bates, 1998), so neuropsychological batteries that do not include tasks tapping emotion-based or motivationally salient aspects of EF may be less sensitive to detecting associations with socio-relational functioning. Moreover, several studies reporting links between EF and socio-emotional skills rely on longitudinal designs, in which EF predicts later socio-relational competencies (Moffitt et al., 2011). Finally, although we examined risk factors in the three developmental domains separately to provide clear and domain-specific interpretations, it is well known that these areas of development are highly interrelated. Even though interaction effects between different types of early risk were not statistically significant, inspection of individual cases revealed that difficulties in motor, language, and socio-relational domains often co-occurred within the same children.

From a clinical perspective, these findings may inform early screening and preventive practices by highlighting developmental markers that could be associated with later executive functioning. Systematic monitoring of early gross- and fine-motor milestones, as well as morpho-syntactic language development, may therefore contribute to a more nuanced developmental evaluation. For example, persistent delays in locomotor abilities, fine-motor coordination, or in the correct use of temporal terms and early written name production may represent potential indicators of vulnerability in certain executive domains and could warrant closer developmental observation. Within a developmental cascade framework, early vulnerabilities in motor or language domains may be understood as factors that interact over time with emerging executive processes, suggesting that supportive language-based or motor-enrichment activities might be considered as part of a broader preventive perspective.

Beyond their clinical implications, it is also worth noting some methodological features of the present study. A strength of this study lies in its multi-method design: data were gathered from different sources. The parent-report questionnaire on early and current development provided ecological information similar to what clinicians typically receive during an initial intake with a child’s family, whereas the performance-based tasks administered by trained examiners yielded data on children’s executive functioning that are less susceptible to informant bias. Another point of originality concerns the sample characteristics. The sample consisted of children likely to be typically developing, with CPM scores within the normal range and without any diagnosed neurodevelopmental disorder at the time of data collection; nonetheless, part of this group displayed early motor and/or language risk factors. In the existing literature, most retrospective studies examining the influence of early developmental milestones on EF or self-regulation have relied on samples of children diagnosed with ADHD or ASD (Gurevitz et al., 2012; Johnson et al., 2015).

### Limitations and Future Directions

Among the limitations of the present study is the fact that it was not possible to control for the influence of early executive abilities on motor, language, and socio-relational development. Therefore, although the findings provide useful indications regarding potential early warning signs to consider during anamnesis and for preventive purposes, they cannot offer stable evidence regarding the directionality of the investigated relationships.

It is also important to highlight, as noted by Benso (2013), that specific tasks are often assumed to tap specific cognitive functions. The tasks included in the battery inevitably involve a combination of executive and non-executive abilities (e.g., perceptual or linguistic skills). However, the use of CFA allowed us to more confidently isolate the variance attributable to the cognitive function most strongly elicited by each task, strengthening the interpretation of the associations observed.

Another limitation concerns the substantial number of missing responses in the PODS-Q, which—together with study requirements (e.g., Raven’s Colored Progressive Matrices scores above the 15th percentile)—led to a considerable reduction in the final sample size compared to the initial pool (*N* = 416). In this regard, the specific sampling strategy adopted may also limit the representativeness of the sample. Because children below the 15th percentile on Raven’s matrices were excluded and only a proportion of non-risk children was randomly selected, the observed variability may not fully reflect the natural developmental distribution in the general population. Accordingly, the generalizability of the findings should be interpreted with caution. Moreover, because the battery was administered across different preschool settings, contextual factors such as the presence of a teacher in the room, environmental distractions, and children’s fatigue at different times of the day may have affected performance on some tasks.

A further limitation concerns the retrospective nature of the early developmental data collected through the PODS-Q. Parents were asked to recall their child’s motor, language, and socio-relational milestones during the first three years of life. Although the questionnaire focuses on salient developmental milestones that are generally well remembered (e.g., walking, first words), retrospective reporting may be subject to recall bias, particularly given that the time interval between the early developmental period and assessment varied across participants. Such inaccuracies are likely to introduce random measurement error, potentially attenuating associations rather than inflating them. Future studies employing prospective longitudinal designs would strengthen the validity of early developmental assessments.

Finally, the relatively wide age range of the sample (3–6 years) should be considered, as executive functions undergo rapid developmental changes during this period. Age-related variability may therefore have contributed to differences observed across measures.

Beyond increasing the sample size, future research should consider following children over a longer period or implementing a longitudinal design, incorporating objective measures of attentional and executive abilities as well as motor, linguistic, and socio-relational competences during the early years of life. Such an approach would allow researchers to examine the interplay between different areas of cognitive development without losing sight of the overall developmental picture, an aspect that is fundamental for clinical assessment and planning.

## 5. Conclusions

This study examined whether early motor, language, and socio-relational development predicts preschool EF in a sample of 110 children. Parents completed the PODS-Q questionnaire, which provided information on children’s skills at 6–36 and 37–72 months, while EFs were assessed through a neuropsychological battery. CFA supported a two-factor structure—WM-CF and IC—and these factor scores were used as dependent variables in subsequent analyses. The results showed that early language skills, particularly grammatical abilities, were significant predictors of WM-CF, whereas early gross-motor abilities were significant predictors of IC. These findings align with a developmental-cascade framework, suggesting that even mild early delays in motor or language development may reduce opportunities for exploration, interaction, and learning processes that scaffold EF growth. Although socio-relational milestones did not significantly predict EF outcomes, this may reflect the adaptive nature of the socio-relational subscale used and the fact that the battery did not include hot EF tasks specifically tapping socio-emotional processing, as supported by previous research. Qualitative patterns nonetheless indicate that vulnerabilities across developmental domains often co-occur, even when statistical interactions are not detected. Overall, these results highlight the clinical relevance of monitoring early developmental milestones to identify children who may be at increased risk of later executive difficulties. Early identification may support more timely assessments and preventive interventions, promoting core executive abilities during a period of heightened neurodevelopmental plasticity.

## Figures and Tables

**Table 1:** Description of the study sample.

	At Risk 3–6 Years	Not at Risk 3–6 Years	Total
At risk 6–36 months	*n* = 24	*n* = 29	*n* = 53
Not at risk 6–36 months	*n* = 4	*n* = 53	*n* = 57
Total	*n* = 28	*n* = 82	*N* = 110

**Table 2:** Descriptive statistics for performance on Executive Function tasks.

Neuropsychological Tasks	*M*	*DS*	Skewness	Kurtosis	*N*
Color and Shape Game (0–36)	18.09	4.836	−1.73	0.45	110
Day/Night Stroop (0–16)	2.47	3.996	1.72	2.90	108
Truck loading (0–8)	4.00	2.893	0.16	−1.51	110
Statue (0–45)	3.94	5.472	2.35	6.16	106
Digit Span Forward (0–16)	4.92	2.132	−0.10	0.23	108
Digit Span Backward (0–16)	1.93	2.109	0.70	−0.60	107
The Gift (0–120)	102.49	38.30	0.16	−1.51	107

**Table 3:** Regression of early and late developmental risk factors (motor, language, socio-relational) on executive functions.

	Inhibitory Control				Working Memory–Flexibility			
	β	*SE*	*t*	*p*	β	*SE*	*t*	*p*
Early Motor Risk	−0.255	0.081	−2.652	009	−0.120	0.115	−1.241	.217
Late Motor Risk	−0.082	0.089	−0.854	395	−0.207	0.126	−2.141	.035
Early Language Risk	−0.240	0.077	−2.518	013	−0.267	0.104	−2.899	.005
Late Language Risk	−0.073	0.093	−0.767	445	−0.194	0.125	−2.105	.038
Early Socio-Relational Risk	−0.155	0.081	−1.533	128	−0.122	0.113	−1.206	.230
Late Socio-Relational Risk	−0.011	0.097	−0.111	912	−0.071	0.135	−0.698	.487

## Data Availability

The data presented in this study are available on request from the corresponding author. The data are not publicly available due to privacy restrictions.

## Abbreviations

The following abbreviations are used in this manuscript:

EF	Executive Function
IC	Inhibitory Control
WM	Working Memory
CF	Cognitive Flexibility
ASD	Autism Spectrum Disorder
CFA	Confirmatory factor analysis
ADHD	Attention Deficit Hyperactivity Disorder
MANOVA	Multivariate Analysis of Variance
